# Development and validation of the information ethics behavior evaluation scale for nurses

**DOI:** 10.5116/ijme.68d1.220a

**Published:** 2025-09-30

**Authors:** Hitomi Sakamoto, Takuya Nagamine

**Affiliations:** 1 Department of Nursing Science, Faculty of Nursing and Nutrition, University of Nagasaki, Japan

**Keywords:** Informational ethics, nursing ethics, ethical behavior

## Abstract

**Objectives:**

This study aimed to develop a reliable and valid scale to assess nurses’ information ethics behavior, facilitating self-reflection and supporting the application of ethical principles in clinical settings involving digital information management.

**Methods:**

A scale development study with exploratory factor analysis was conducted in 2023, targeting 1,464 hospital-based and home-visit nurses across Japan. Participants completed a preliminary version of the Information Ethics Behavior Evaluation Scale for Nurses along with demographic questions. Item analysis and exploratory factor analysis (EFA) using the alpha factor extraction method and promax rotation were performed. Criterion-related validity was assessed via Spearman’s rank-order correlation with the Self-Evaluation Scale for Ethical Behavior as a Nurse.

**Results:**

Valid responses were obtained from 427 participants. Item analysis led to the exclusion of 14 items due to low factor loadings or double loading. EFA identified a three-factor structure comprising 21 items: (1) conscious behavior in handling information, (2) appropriate information management, and (3) response to information leakage risk. The scale demonstrated strong internal consistency (Cronbach’s α = .86). Criterion-related validity was supported by a significant correlation with the external measure (r_s_ = .74, p < .001).

**Conclusions:**

The Information Ethics Behavior Evaluation Scale for Nurses is a brief, reliable, and valid tool for assessing ethical conduct related to information handling in nursing practice. It provides a framework for ethical self-assessment and may contribute to the prevention of information breaches and the promotion of ethical decision-making. Further research should investigate the integration of digital literacy and internet-specific ethical competencies.

## Introduction

Nursing ethics, like medical ethics, addresses key ethical issues in patient–provider relationships, including autonomy, informed consent, truth‑telling, and confidentiality. With the rapid advancement of information technology and increasing complexity in healthcare data management, ethical considerations in nursing information handling have reached a critical juncture. Patient information is now routinely stored in electronic health records and cloud-based systems, making it more susceptible to data breaches and cyberattacks that threaten confidentiality.^1^

Although real‑time updating and sharing of patient data enhance nursing efficiency, errors in data entry or interpretation can significantly impact patient outcomes. The expanding use of artificial intelligence (AI) for predicting patient conditions and supporting diagnosis has raised concerns, particularly regarding its potential to prioritize efficiency over patient autonomy or to compromise patients’ human rights due to a treatment‑centered approach. Furthermore, the pace of technological innovation far outstrips the development of regulatory frameworks in many countries, suggesting that reliance solely on laws or procedural guidelines is insufficient.^2^ Ethical competence—which encompasses the ability to recognize ethical issues through heightened moral sensitivity and to act appropriately—is therefore essential.^3^ Such competence is not innate or acquired immediately upon certification, but develops with experience and reflection.

Shinagawa et al. reported that, between April 2005 and May 2017, the most frequent causes of publicly reported information leakage incidents by nurses included loss or misplacement of data (36.8%), unauthorized extraction (27.9%), and improper transmission (20.6%).^4^ These findings indicate that breaches often result from ethical lapses rather than technical incompetence. Aoyagi noted that a "sense of responsibility for one’s role in patient‑centered care " is a core component of ethical sensitivity among healthcare professionals.^5^ Even absent tangible harm, the erosion of patient trust following breaches of personal information can be profound.

While modern technology streamlines nursing workflows, nurses still must safeguard patient privacy, ensure accuracy of documentation, and make ethically informed decisions in increasingly complex ICT environments. To support this goal, the concept of technologizing morality has been proposed—embedding ethical behavior into systems so that moral practice is supported by design rather than reliant solely on individual cognition.^6^ Ultimately, the aim of ethical development in nursing is not superficial moralism but strengthening practical decision‑making grounded in humanistic care values.^7^^, ^^8^

Yet, delaying nurses' engagement with information systems until full ethical competence is achieved is unrealistic. Instead, ethical growth is fostered through active practice. In response, we developed the Information Ethics Behavior Evaluation Scale for Nurses—a concise tool designed to facilitate self‑reflection and embed ethical behavior in routine information handling.

Existing instruments—such as the Ethical Behavior Self‑Evaluation Scale for Nurses^9^ and the Ethical Behavior Scale for Nurses^10^—address general ethics but lack specific focus on information ethics. Therefore, a tailored scale is required. The aim of this study was to evaluate the reliability and validity of the newly developed Information Ethics Behavior Evaluation Scale for Nurses. This tool is intended to support nurses in using information and communication technology with heightened moral sensitivity and ethical judgment, to reduce daily incidents of personal information leakage, and to facilitate ethical self‑assessment in clinical practice. We anticipate that it will contribute to the development of nursing professionals committed to ethical practice in digital healthcare environments.

## Methods

### Study design and participants

We conducted a scale‑development study using exploratory factor analysis, following Oshio’s methodological framework.^11^ This study received approval from the General Research Ethics Committee of the University of Nagasaki. Survey invitations and explanatory materials were mailed to nursing administrators at general hospitals and home nursing stations across Japan. From consenting institutions, participant pools were recruited via stratified random sampling: 20% of hospitals (n = 763) and 20% of home‑visit nursing stations (n = 701) were selected across eight geographic regions. The purpose and methods of the study were explained in writing to nursing directors and managers via a research cooperation request document enclosed with the survey. Returning the completed questionnaire was considered as consent to participate.

Consistent with Loewen and colleagues we aimed for a retest sample constituting at least 10% of the valid responses collected in the baseline survey.^12^ Data collection occurred between February 2022 and July 2023, encompassing initial distribution and follow‑up (retest) surveys. Among the returned surveys, a total of 444 responses were obtained (response rate = 30.3%), of which 427 (29.2%) were valid. Among the valid responses, 349 were from hospital nurses and 78 from visiting nurses (see Table 1).

### Scale development

Information ethics for this study was defined as the ethical handling of nursing data—especially collection and management of core nursing activities (assessment, diagnosis, goal setting, intervention, evaluation)—in accordance with professional standards and patient-centered care frameworks.^13^^,^^14^ Using qualitative analysis of interviews with nursing managers from hospitals and home‑care settings, we drafted a 40‑item pool.^15^ Content validity was established by review from multiple experts—including nursing managers, faculty experienced in scale development, and a nursing informatics specialist—following Polit & Beck’s guidelines.^16^ A pretest was then conducted with seven practicing nurses to assess item clarity and redundancy; four problematic items were revised or removed. Items were standardized to the present tense, yielding a preliminary 36‑item version rated on a four‑point scale (4= Always to 1= Almost never).

### Validity and reliability testing

Criterion‑related validity was assessed by examining Spearman’s rank-order correlations between the new scale and the Self-Evaluation Scale for Ethical Behavior as a Nurse developed by Nagano et al. (2012), with permission. This instrument consists of 31 items rated on a four-point Likert scale.^9^ Internal consistency was evaluated with Cronbach’s Alpha. Stability over time was tested via test–retest: a secondary survey was administered two months after the initial, and agreement was measured using weighted Cohen’s kappa due to non-normal score distributions (confirmed via Shapiro–Wilk test).^17^

### Data collection and analysis

Survey packages—including questionnaires and return envelopes—were distributed and collected via collaborating institutions. Participant demographics included years of nursing experience, location, hospital bed count or patient load (for home‑care nurses), job titles, and use of electronic medical records. Statistical analysis was conducted using IBM SPSS Statistics 28.0, Amos 26.0 Graphics, and HAD version 18.

**Table1:** Basic attributes of the participant (N=427)

Participants		Hospital Nurse	Visiting Nurse	Total
		349	78	427
		Mean	Mean	
Years of clinical nurse experience		19.64	18.82	
Years of experience as a visiting nurse			8.32	
		n	n	n (％)
Location of hospitals and offices				
	Hokkaido	60	4	64 (15.0)
	Tohoku	24	2	26 (6.1)
	Kanto	96	3	99 (23.2)
	Chubu	66	19	85 (19.9)
	Kinki	33	15	48 (11.2)
	Chugoku	4	22	26 (6.1)
	Shikoku	6	7	13 (3.0)
	Kyushu-Okinawa	56	5	61 (14.3)
	Not answered	4	1	5 (1.2)
Number of hospital beds				
	20 - 99 beds	31	-	
	100 - 199 beds	91	-	
	200 - 399 beds	151	-	
	400 - 599 beds	53	-	
	Over 600 beds	16	-	
	Not answered	7	-	
Number of users in charge				
	3 people or less	-	8	
	4 - 10 people	-	21	
	11 - 20 people	-	21	
	21 - 30 people	-	11	
	31 people or more	-	2	
	Not answered	-	15	
Job titles				
	nursing manager	8	22	
	head nurse	54	-	
	chief nurse	114	-	
	nursing staff	173	54	
	Not answered		2	
Whether to introduce electronic medical records				
	introduced	321	64	385 (90.2)
	Not introduced	27	10	37 (8.7)
	Not answered	1	4	5 (1.2)

Item analysis comprised item–total and item–item correlation, good–poor analysis (dividing respondents into top and bottom 33% for t‑tests at α = .05), The Kaiser–Meyer–Olkin (KMO) sampling adequacy, and Bartlett’s test of sphericity. Parallel analysis determined the appropriate number of factors. Exploratory factor analysis employed the alpha factor extraction method with promax rotation. Ceiling effects were not assessed due to anticipated high-frequency Always responses. Spearman’s correlation assessed criterion validity at scale and subscale levels. Cronbach’s Alpha calculated internal consistency. Test–retest reliability utilized weighted kappa.

A confirmatory factor analysis on an independent sample is planned to further validate the factor structure. In the future, we plan to conduct a Confirmatory Factor Analysis on a different sample than the exploratory factor analysis (EFA) conducted this time to statistically verify whether each question item measures the concept as expected.

## Results

### Item analysis

No items exhibited floor effects. In the item–total correlation analysis, Item 1 showed a low correlation with the total score (r = .27, p < .001), which did not meet the commonly accepted threshold of r ≥ .30. Although no items met the exclusion criteria in the good–poor (G–P) analysis, Item 1 was excluded based on the item–total correlation result. Consequently, 35 items were retained for the subsequent EFA.

### Exploratory Factor Analysis

An EFA was conducted on the remaining 35 items using the alpha factoring method with promax rotation. The number of factors was determined through parallel analysis, which indicated a three-factor solution. The KMO measure confirmed sampling adequacy with a value of .91, and Bartlett’s test of sphericity was statistically significant, χ² (210, N = 427) = 3,088.17, p < .001, indicating the suitability of the data for factor analysis.

Following item reduction based on factor loadings below .40 or cross-loadings, 14 items were excluded. The final version of the scale consisted of 21 items and demonstrated good internal consistency, with an overall Cronbach’s Alpha of 0.86. The three extracted factors together explained 48.11% of the total variance, with Factor 1 accounting for 33.29%, Factor 2 for 7.64%, and Factor 3 for 7.18%.

Factor 1, labeled “Conscious behavior in handling information”, included 10 items and showed the highest internal consistency (Alpha = 0.89), reflecting nurses’ awareness and sensitivity regarding ethical use of patient information. Factor 2, “Appropriate information management”, consisted of 5 items (Alpha = 0.71), describing specific practices such as data backup and password security. Factor 3, “Response to information leakage risk”, contained 4 items and demonstrated acceptable reliability (Alpha = 0.65), focusing on compliance with institutional protocols and risk- averse behavior.

A detailed summary of the factor structure, item content, and factor loadings is presented in Table 2.

### Criterion-Related Validity

Criterion-related validity was examined by calculating Spearman’s rank-order correlation between the total score of the 21-item Information Ethics Behavior Evaluation Scale for Nurses and the total score of the Self-Evaluation Scale for Ethical Behavior as a Nurse. The analysis revealed a strong positive correlation (rₛ = .74, p < .001), indicating that the new scale is consistent with an established measure of ethical behavior in nursing.

Each of the three subscales also demonstrated statistically significant correlations with the external measure. The first factor, “Conscious behavior in handling information,” showed the strongest correlation (rₛ = .78, p < .001), suggesting a high degree of alignment with broader ethical competencies. The second factor, “Appropriate information management,” exhibited a moderate correlation (rₛ = .52, p < .001), while the third factor, “Response to information leakage risk,” exhibited a weaker, yet still moderate, correlation (rₛ = .39, p < .001). These results support the concurrent validity of the scale and highlight that each factor captures different aspects of ethical behavior related to information handling.

### Test–Retest Reliability

To assess the stability of the scale over time, test–retest reliability was evaluated. Of the initial participants, 75 completed both the first and second administrations of the scale. After excluding one incomplete response, data from 74 participants (17.3% of the total valid sample) were included in the analysis.

Weighted Cohen’s kappa coefficient was used to assess the level of agreement between the two time points. The total scale score demonstrated moderate reliability, with a kappa value of .52 (p < .001). When analyzed by subscale, the first factor showed moderate agreement (κ = .57, p < .001), the second factor showed fair agreement (κ = .43, p < .001), and the third factor again showed moderate agreement (κ = .59, p < .001). These findings indicate that the scale yields reasonably consistent results over time, particularly for the factors most closely associated with habitual and reflective ethical behaviors.

## Discussion

### Reliability and Validity of the Scale

This study developed a 21-item, three-factor Information Ethics Behavior Evaluation Scale for Nurses, based on a 36-item draft derived from prior research. Internal consistency was evaluated using Cronbach’s Alpha. The total scale demonstrated good reliability with Alpha = 0.86. However, the Alpha coefficient for the third factor was Alpha = 0.65. According to Taber’s interpretation, this level is considered satisfactory but still warrants further refinement.^18^

Test–retest reliability was assessed using weighted Cohen’s κ, which yielded κ = .52. This is categorized as moderate reliability based on Landis and Koch’s benchmarks. These results suggest that the scale demonstrates acceptable reliability, though further validation is desirable.^19^

For criterion-related validity, Spearman’s rank correlation was calculated between the total score on this scale and the Self-Evaluation Scale of Ethical Behavior as a Nurse. A significant positive correlation was observed, supporting the concurrent validity of the scale. However, the third factor showed a weaker correlation (rₛ = .39, p < .001). This may be due to the factor’s emphasis on the "technologization of morality"—items that describe procedural compliance, such as refraining from posting patient-related content on social media, rather than internalized ethical reasoning.

**Table 2:** Exploratory Factor Analysis of nursing information ethics behavior evaluation scale（N=427)

♯	Items	Factor loading		
		1	2	3
Factor1: Conscious behavior in handling information [Cronbach’s α = 0.89]				
q11	Build a relationship of trust with patients (users) and avoid words and actions that may damage the relationship of trust or one-sided communication on the part of nurses	.741	-.143	.125
q16	Raise awareness of handling personal information as a nursing professional, be aware of information risk management, and act with self-awareness	.687	.112	-.036
q12	Respect the patient's (user's) wishes when handling information, and be considerate of information that the patient (user) may feel embarrassed about or may not want others to know	.678	-.166	.099
q28	Make the most of the information obtained and apply it to individualized nursing practice	.658	.078	-.167
q14	Provide information as needed by the patient (user) and information deemed necessary by the nurse according to the patient's (user's) medical condition and life background in an appropriate manner, and support the patient (user) in decision making	.621	.029	-.024
q25	Ensure accuracy when recording and sharing information, and strive to ensure that the truth is conveyed without excess or deficiency, regardless of intent or negligence	.576	.150	-.050
q22	Respect the human rights, beliefs, and will of the patient (user) to the greatest extent possible, and take care to avoid the use of information by the nurse or intentional manipulation of information	.569	.169	.056
q18	When sharing patient (user) information with multiple professions, the necessity and content of the information to be shared should be carefully examined, and the sharing should be done in a timely manner	.566	.171	-.039
q35	Act to protect the personal information and privacy of patients (users) and their families in accordance with the Personal Information Protection Law	.559	.173	.064
q34	As a professional, adhere to the “Code of Ethics for Nursing Professionals” and be highly aware that you are handling personal information	.532	.184	.042
Factor2: Appropriate information management [Cronbach’s α = 0.71]				
q32	Do not use software or applications that are known to be vulnerable, and upload security software as needed to prevent information leakage	.101	.615	.003
q21	When it is necessary to enter personal information, set a password and change the password regularly to properly manage the information	-.199	.547	.274
q29	Take measures to ensure that individuals cannot be identified when sending information	.119	.534	.072
q26	Lock data and documents that contain or store personal information	.159	.457	-.134
q30	Always log off or close the screen when leaving the electronic medical record or computer, and do not leave paper medical records or documents open for others to see	.233	.446	-.048
Factor3: Responding to Information Leakage Risks [Cronbach’s α = 0.65]				
q10	Do not show medical records to third parties without the consent of the patient (user) himself/herself or his/her family, regardless of whether it is beneficial or detrimental to the patient (user) in question, except when “in accordance with laws and regulations” under the Personal Information Protection Law	.026	-.089	.521
q6	Not to access electronic medical records unnecessarily, nor to view documents containing paper medical records for the purpose of interest	.366	-.193	.457
q24	Do not upload patient (user) information or information about the hospital or office to social networking sites	-.153	.246	.451
q5	Observe confidentiality obligations, do not discuss information obtained outside of work, and do not raise topics of interest	.303	-.095	.450
q3	Comply with hospital and office manuals on medical information systems and information management	-.030	.255	.407
q2	Do not use personal e-mail accounts, IDs, or information terminal devices when handling patient (user) information	-.093	.227	.401
	Percentage of total variance explained by initial eigenvalues	33.29	7.64	7.18
	Inter-factor correlation			
	Factor1	-	.53	.54
	Factor2	-	-	.31
	Factor3	-	-	-

Note: An exploratory factor analysis with an alpha factoring method and promax rotation.

### Components of the Scale

The scale consists of three conceptual dimensions. Despite the relatively low reliability for the third factor, “Response to the risk of information leakage,” its conceptual significance warrants attention. This factor aligns with Ode’s subscale on "risk aversion," which falls under the bioethical principle of non-maleficence and includes adherence to confidentiality and information security practices.^10^

There is an inherent risk in managing patient data; however, the professional judgment of nurses often justifies its handling, as the benefit to patient care outweighs potential harm. Personal information is essential for making accurate assessments and diagnoses. Thus, ethical risks are not solely defined by technical properties but also by human behavior and social structures.^20^ Items in this factor, such as "prohibition of using personally owned devices" and "compliance with institutional manuals," align with risk avoidance strategies. Yet, adherence to these rules may conflict with operational efficiency.

Additionally, the inclusion of items related to “interest-oriented” behavior—such as using devices for personal gain—resonates with Murray’s framework on ethical desire and motivation. Embedding ethical norms into routine behavior through system design—i.e., "technologizing morality"—may enable consistent ethical conduct even when conscious deliberation is absent. Such habituation is expected to foster "conscious behavior in handling information" and enhance ethical decision-making skills over time.

### Implications for Practice

The revised Guidelines for the Safety Management of Medical Information Systems issued by Japan’s Ministry of Health in 2023 highlight the growing urgency of cybersecurity in healthcare.^21^ In the same year, the National Police Agency reported 230 ransomware incidents, including 20 cases involving medical or welfare institutions.^22^ While the number of cases may seem relatively low, the financial impact can be substantial—estimated at ¥50–100 million (approximately GBP 270,000–540,000) per incident. In addition to monetary losses, victims may experience reputational damage and disruptions in the delivery of medical services.^23^ To counter these threats, emergency action planning and training are critical, yet technical safeguards remain insufficient. Raising awareness of password management, antivirus protection, and resistance to targeted cyberattacks is equally necessary.^24^^, ^^25^

The second factor, “Appropriate information management,” includes behaviors such as updating security software, managing passwords effectively, and backing up data. These practices reflect ethically appropriate responses to the risk of data leakage.

In nursing, ethical decision-making should focus on anticipated outcomes.^26^ Decisions must be made in context—based on timing, environment, and individual values—and are likely to change upon deeper reflection. The first factor, “Conscious behavior in handling information,” captures this dynamic. It includes abstract attitudes like high awareness of personal information handling, proactive privacy protection, and sensitivity toward information others may wish to keep private. Such awareness should be integrated into a variety of nursing scenarios, guiding ethically sound actions.

Importantly, the value of information lies not merely in its confidentiality but in its meaningful application.^27^ Utilizing patient data effectively and ethically to support individualized care reflects both moral responsibility and professional identity in nursing.

### Limitations

During the scale development process, items were selected based on ethical behaviors identified in previous research. As a result, certain aspects of information ethics behavior may not be fully captured by this scale. Future research should conduct confirmatory factor analysis (CFA) using an independent sample to further validate the factor structure and improve the generalizability of the findings.

Additionally, a previous study reported that individuals with strong moral awareness can still cause problems due to a lack of understanding regarding the characteristics of the internet.^28^ Unlike general ethical conduct, information ethics involves complex interactions between ethical principles and fundamental digital knowledge. Future studies should incorporate dimensions of digital literacy and information ethics literacy to address this important aspect of ethical behavior in digital environments.

## Conclusions

The 21-item Information Ethics Behavior Evaluation Scale for Nurses developed in this study comprises three subscales: Conscious behavior in handling information, Appropriate information management, and Response to information leakage risk. The scale demonstrated acceptable validity and reliability.

This tool enables nurses to reflect on and evaluate their own practices in managing nursing-related information and may serve as an effective framework for fostering ethical decision-making in clinical settings. By supporting ethical competence in digital information handling, the scale has the potential to contribute to improved information security and professional accountability in nursing.

Implications for medical education include incorporating information ethics as a core component of nursing and healthcare curricula. Given the growing reliance on digital systems in clinical care, educational programs should provide structured opportunities for students to develop ethical decision-making skills specifically related to information management. The scale developed in this study may serve as a practical educational resource for cultivating ethical awareness, encouraging self-reflection, and fostering professional responsibility among nursing students and early-career clinicians.

Future research should aim to validate the scale in diverse clinical and cultural contexts, and investigate its effectiveness when used as part of ethics education programs. Longitudinal studies are also recommended to examine how the scale contributes to the ongoing development of nurses’ ethical competence over time.

### Acknowledgments

We thank the nurses who participated in this study. This study was supported by a grant from the President's Discretionary Research Fund of University of Nagasaki in 2023.

### Conflict of Interest

The authors declare that they have no conflict of interest.
